# Immunohistochemical Expression of Five Protein Combinations Revealed as Prognostic Markers in Asian Oral Cancer

**DOI:** 10.3389/fgene.2021.643461

**Published:** 2021-04-15

**Authors:** Hui-Ching Wang, Chien-Jung Chiang, Ta-Chih Liu, Chun-Chieh Wu, Yi-Ting Chen, Jan-Gowth Chang, Grace S. Shieh

**Affiliations:** ^1^Graduate Institute of Clinical Medicine, College of Medicine, Kaohsiung Medical University, Kaohsiung, Taiwan; ^2^Division of Hematology and Oncology, Department of Internal Medicine, Kaohsiung Medical University Hospital, Kaohsiung Medical University, Kaohsiung, Taiwan; ^3^Institute of Statistical Science, Academia Sinica, Taipei, Taiwan; ^4^Department of Hematology-Oncology, Chang Bing Show Chwan Memorial Hospital, Changhua, Taiwan; ^5^Department of Pathology, Kaohsiung Medical University Hospital, Kaohsiung Medical University, Kaohsiung, Taiwan; ^6^Epigenome Research Center, China Medical University Hospital, Taichung, Taiwan; ^7^Department of Laboratory Medicine, China Medical University Hospital, Taichung, Taiwan; ^8^Center for Precision Medicine, China Medical University Hospital, Taichung, Taiwan; ^9^School of Medicine, China Medical University, Taichung, Taiwan; ^10^Department of Bioinformatics and Medical Engineering, Asia University, Taichung, Taiwan; ^11^Bioinformatics Program, Taiwan International Graduate Program, Academia Sinica, Taipei, Taiwan; ^12^Genome and Systems Biology Degree Program, Academia Sinica and National Taiwan University, Taipei, Taiwan; ^13^Data Science Degree Program, Academia Sinica and National Taiwan University, Taipei, Taiwan

**Keywords:** biomarker, cox regression, immunohistochemistry, oral cancer, overall survival, prognosis, gene expression data

## Abstract

Oral squamous cell carcinoma (OSCC) has a high mortality rate (∼50%), and the 5-year overall survival rate is not optimal. Cyto- and histopathological examination of cancer tissues is the main strategy for diagnosis and treatment. In the present study, we aimed to uncover *immunohistochemical* (IHC) markers for prognosis in Asian OSCC. From the collected 742 synthetic lethal gene pairs (of various cancer types), we first filtered genes relevant to OSCC, performed 29 IHC stains at different cellular portions and combined these IHC stains into 398 distinct pairs. Next, we identified novel *IHC* prognostic markers in OSCC among Taiwanese population, from the single and paired IHC staining by univariate Cox regression analysis. Increased nuclear expression of RB1 [RB1(N)↑], CDH3(C)↑-STK17A(N)↑ and FLNA(C)↑-KRAS(C)↑were associated with survival, but not independent of tumor stage, where C and N denote cytoplasm and nucleus, respectively. Furthermore, multivariate Cox regression analyses revealed that CSNK1E(C)↓-SHC1(N)↓ (*P* = 5.9 × 10^–5^; recommended for clinical use), BRCA1(N)↓-SHC1(N)↓ (*P* = 0.030), CSNK1E(C)↓-RB1(N)↑ (*P* = 0.045), [CSNK1E(C)-SHC1(N), FLNA(C)-KRAS(C)] (*P* = 0.000, rounded to three decimal places) and [BRCA1(N)-SHC1(N), FLNA(C)-KRAS(C)] (*P* = 0.020) were significant factors of poor prognosis, independent of lymph node metastasis, stage and alcohol consumption. An external dataset from The Cancer Genome Atlas HNSCC cohort confirmed that *CDH3*↑-*STK17A*↑ was a significant predictor of poor survival. Our approach identified prognostic markers with components involved in different pathways and revealed IHC marker pairs while neither single IHC was a marker, thus it improved the current state-of-the-art for identification of IHC markers.

## Introduction

Head and neck squamous cell carcinoma (HNSCC) is the sixth most common cancer globally (Bray et al., 2018). Every year, more than 700,000 new cases of HNSCC are diagnosed and 350,000 related deaths are reported worldwide. Oral squamous cell carcinoma (OSCC) is the most common cancer of the head and neck region and has a high mortality rate. However, little improvement has been made in the five-year overall survival rate over the years (Bray et al., 2018). Identifying reliable prognostic factors remains challenging. OSCC is believed to originate from the multistep accumulation of heterogeneous genetic changes in squamous cells. These changes progressively enable transformed cells to proliferate and invade (Oliveira and Ribeiro-Silva, 2011). These accumulated changes may explain why tumors at the same clinical stage and localization often show significant differences in clinical outcome.

The main causes of OSCC in Taiwan and some South Asian countries (Belcher et al., 2014) are the consumption of alcohol, tobacco, and betel nut. This contrasts with human papillomavirus (HPV)-positive oropharyngeal SCC which is associated with HPV infection, with higher proportions in western populations than Asian populations (Gillison et al., 2000).

Unlike other malignancies, the relationships between mutations of genes and clinical morphological characteristics such as tumor grade in OSCC are obscure, which has impeded the development of personalized medicine. Cytopathological and histopathological examination of cancer tissues remains the main diagnostic and treatment strategy for OSCC. Although immunohistochemical (IHC) staining may be limited by small volumes taken from samples, varying expression with selected antibodies, and partial reliance on subjective perception, IHC staining provides morphological information about protein expression, and it is simple and cost-effective. Moreover, the procedures and guidelines (Wolff et al., 2007; Hammond et al., 2010; Dowsett et al., 2011) for IHC staining are well established, and widely used in clinics.

The primary aim is to identify a panel of IHC prognostic markers for Asian OSCC, to enable the selection of patients best suited for intensive adjuvant therapy in clinics. Most of previous results on IHC prognostic markers in OSCC were mainly based on one protein, few on two proteins or on one pathway and are reviewed briefly as follows. IHC of cyclin D1, MDM2, and γ-catenin were shown to be potential prognostic markers in a study of 55 patients with buccal SCC who regularly chewed betel nut (Peng et al., 2011). In 2005, IHC of cyclin D1 and Rb overexpression combined with p16 underexpression (denoted by cyclin D1↑-Rb↑p16↓) (Jayasurya et al., 2005) and Rb↓–p53↑ (Soni et al., 2005) were shown to be associated with poor prognosis in a cohort of 348 and a cohort of 98 Indian patients with OSCC, respectively. Moreover, simultaneous coexpression of p53, cyclin D1, and EGFR was a significant prognostic factor in a cohort of 140 Japanese patients with oral cancer (Shiraki et al., 2005). P-cadherin was reported to be marginally significantly associated with poor survival in a small cohort (Muzio et al., 2005). About a decade later, CK1ϵ nonexpression (Lin et al., 2014) and expression of BRCA1 and γH2AX (Oliveira-Costa et al., 2014) were shown to be associated with poor overall and disease-specific survival, respectively.

**FIGURE 1:**
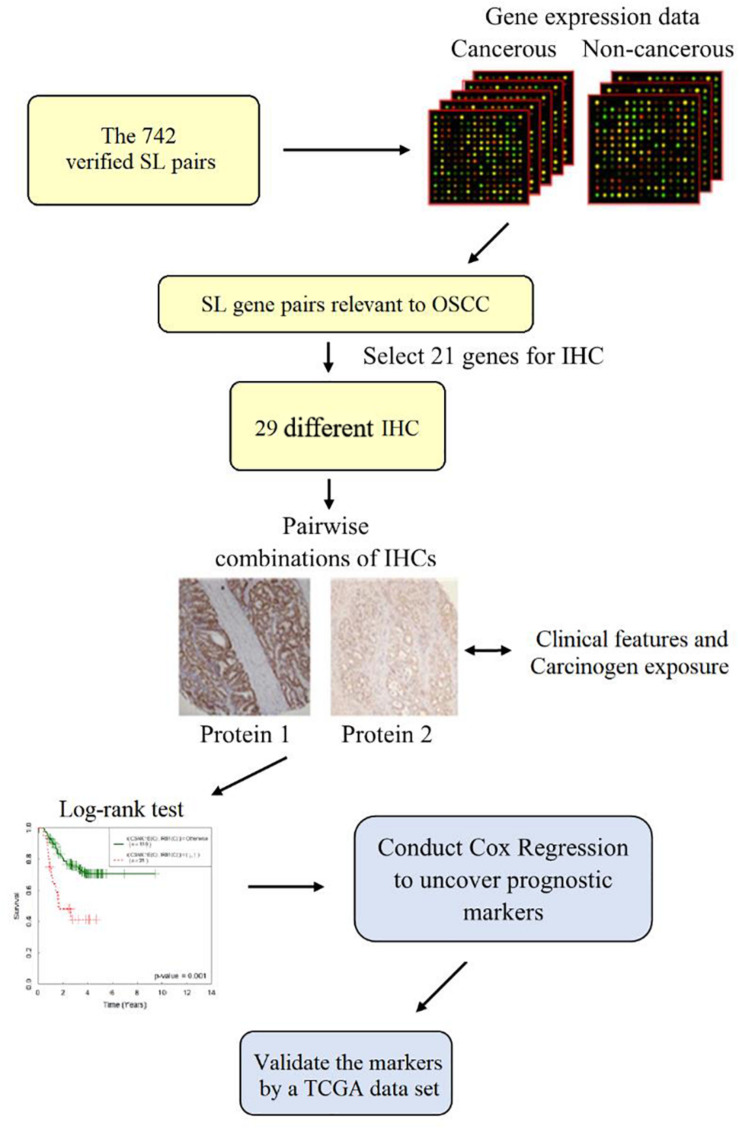
Schematic graph of the study approach. Microarray gene expression of 57 oral cancerous and 22 noncancerous tissues selected OSCC-relevant gene pairs from 742 verified synthetic lethal pairs. Twenty-one genes were marked for immunohistochemistry staining. Pairwise combinations of the 29 IHCs followed by a log-rank test and Cox regression models revealed single/paired and combined prognostic markers.

We started with a list of 742 synthetic lethal (SL) gene pairs collected from the literature, which consisted of several oncogenes, tumor-suppressor genes, genome stability and other cancer genes with important functions. Two genes are termed SL genes if a single mutation of either is not lethal, but their simultaneous mutation leads to cell death (Chang et al., 2016). The SL interactions of these collected pairs in various cancer types are validated either with human cancer cell lines (Bryant et al., 2005; Farmer et al., 2005) or by genome-wide RNA interference (RNAi) knockdown (Barbie et al., 2009; Luo et al., 2009). The list of SL gene pairs can be accessed at^1^. SL pairs are shown to be correlated to survival of cancer cells (Kaelin, 2005). In general, the more cancer cells killed, the better cancer patients’ survival. Thus, we speculated that SL pairs are relevant to prognosis assessment. We hypothesized that IHC (protein) expression is concordant to its gene expression, and we used gene expression data of Asian OSCC to select an initial panel of SL pairs which are relevant to OSCC, from the collected SL pairs (of all types of cancer). Next, we adopted the rule of frequently co-expressed gene pairs along with prior knowledge of OSCC to select ∼20 genes for IHC staining. IHC staining is conducted because protein is more stable than mRNA, ultimately functions in cells, and IHC is usable in the clinic. We also combined single IHC into 398 distinct IHC pairs. To identify prognostic markers, we applied Cox regression analysis to each single IHC (each combined IHC pair) and the overall survival of patients with OSCC. Previously, we applied this approach to colorectal cancer (Chang et al., 2016) and lung adenocarcinoma (Liu et al., 2004), and both studies successfully uncovered IHC prognostic markers independent of tumor stage, in addition to revealing novel IHC marker pairs where neither single IHC was a marker. Our approach starts with the collected SL pairs, which allows components participating in different pathways to be identified as prognostic marker pairs. This improves the current state of the art for IHC marker discovery, which hitherto has mainly relied on one protein, few on two proteins or one pathway (Oliveira-Costa et al., 2014). After the prognostic markers were revealed, we validated them using OSCC data with HPV(−) from The Cancer Genome Atlas (TCGA) HNSCC cohort. A schematic graph of our method is presented in Figure 1.

## Materials and Methods

### Study Population

A total of 163 cases of oral cavity cancers were identified in the Kaohsiung Medical University Hospital. Although this sample size was moderate, it was less than only four of the 20 and more previous studies. Furthermore, we conducted a large scale of IHC study and the sample size was sufficiently large for multivariate Cox regression analysis. The inclusion criteria for this study were as follows: (1) age at diagnosis of 20 years or older; (2) tumor histology of squamous cell carcinoma with grade 1 to grade 3; (3) ICD-9 site code specific for the oral cavity; (4) patients underwent surgical interventions,; and (5) disease was diagnosed between 2012 and 2014. The exclusion criteria were: (1) patients who underwent biopsy without surgery; (2) patients with secondary malignancy; (3) tumor histology of carcinoma *in situ*; and (4) SCC from nasopharynx, oropharynx, hypopharynx, and larynx.

### Statistical Analyses, Tissue Arrays and IHC Staining

In the following, all statistical tests were two-sided except where otherwise specified, and all analyses were conducted in R software (R Core Team, 2019).

### Preprocessing of Gene Expression Profiles for Oral Cavity Cancerous Versus Non-cancerous Tissues

Gene expression datasets were selected based on the following parameters: cancerous and noncancerous tissues, no treatments, no metastasis, and Affymetrix chips (up to November 2010). The OSCC gene profiles conforming to the aforementioned criteria were downloaded from GEO. Mutated genes associated with oncogenesis may differ among various ethnic groups (Ding et al., 2008). Therefore, we collected gene expression data from patients of Han Chinese origin [tissues from patients in Taiwan, GSE 25099 (Peng et al., 2011)], which was the same ethnicity as that of IHC and clinicopathological data used here. Gene expression profiles of the 57 OSCC and 22 noncancerous tissues in the dataset were quantile-normalized using “expresso” in R, then for a given gene the log ratio of its expression in each cancer tissue versus that of the averaged non-cancerous tissues was computed.

### Inference of the Initial Panel of Relevant SL Gene Pairs (Table 2) Using Microarray Gene Expression Data

For each SL gene pair, the fractions of (up, up), (up, down), (down, up), and (down, down) patterns were computed, where the cutoff value for up and downregulation was 1.5-fold. The pattern fractions were computed using the log ratios of the microarray gene expression data for the 57 patients with OSCC (GSE 25099).

### Permutation Test and False-Positive Rates of the Fractions of Paired Gene Expression

To evaluate the statistical significance (P value) of the fractions of (up, up) and (down, up) patterns of each gene pair in Table 2, for each fraction we conducted a permutation test to generate its nonparametric distribution. The total rearrangements of the labels of (57) cancer and (22) noncancerous tissues *wasequalto*$\left(\begin{array}{c} 79\\ 22 \end{array}\right)$, from which we randomly chose 10,000 rearrangements. For each rearrangement, we computed the fraction of a pattern to form its distribution, from which we assessed the P value of an observed fraction. Moreover, we applied the q-value (Storey and Tibshirani, 2003) (“*q* value” in R) to estimate the false discovery rate (FDR) of the significance of the gene pairs in Table 2.

### Selection of Genes From the Initial Panel for IHC Staining

We first selected genes whose fractions of the (up, up) and (down, up) patterns were ≥15%, except ≥25% (more stringent) for the (down, up) pattern of *KRAS* SL pair, because the mutation rate of *KRAS* was only ∼2% in OC and there were 200 and more *KRAS* pairs. Next, we applied prior knowledge to (i) select *CDH3* (the top-3) from the top three partners of *EGFR* and the top-1 [*STK17A*, relevant to OSCC (Pickering et al., 2013)] and top-3 (*CDK6*, a tumor suppressor gene) partners of *KRAS* from the *KRAS* SL pairs satisfying the above fraction cutoffs, and to (ii) include genes whose fractions were on the borderline of 15%; this included *FEN1-RAD54B* [involved in nonhomologous DNA end joining repair pathway (Storey and Tibshirani, 2003); 14%], *RB1* [relevant to OSCC (Liu et al., 2004; Presson et al., 2011); 12%] and *MSH2-POLB* (Kang et al., 2009; Tiong et al., 2014; Chang et al., 2016).

### Tissue Microarray Preparation

Clinicopathological features of 163 OSCC patients were collected (Table 1), and their representative cancer specimens were randomly selected from H&E-stained sections and confirmed by pathologists (Chun-Chieh Wu and Yi-Ting Chen). Three cancerous and one noncancerous tissue cores (diameter 2 mm) were longitudinally cut from each paraffin block. The tissue cores were mounted with fine steel needles in new paraffin blocks to produce tissue microarrays. This study was approved by the Institutional Review Board and Ethics Committee of Kaohsiung Medical University Hospital and the Institutional Review Board of Academia Sinica [Nos. KMUHIRB-E(I)-20170034 and AS-IRB-BM-16075]. The data was analyzed anonymously, and therefore no additional informed consent was required. All methods were performed in accordance with the approved guidelines and regulations and the waiver for the informed consent had been obtained from the approving committee.

**TABLE 1:** Clinicopathological characteristics of the OC patients in the study population.

Study population		
	**KMU (*N* = 163)**	
**Characteristic**	***N***	**%**
Age at diagnosis, year		
≦55	84	51.5
>55	71	43.6
NA^a^	8	4.9
Grade		
Low	67	41.1
Intermediate	85	52.1
High	2	1.2
NA	9	5.5
Stage		
I	66	40.5
II	26	16.0
III	19	11.7
IV	41	25.2
NA	11	6.7
Morphology		
Squamous	163	100.0
T^b^		
T1	74	45.4
T2	42	25.8
T3	11	6.7
T4	28	17.2
NA	8	4.9
N^b^		
N0	115	70.6
N1	24	14.7
N2	16	9.8
NA	8	4.9

*^*a*^NA denotes missing data; ^*b*^T and N denote tumor size and lymph node status of “TNM” (AJCC version 7), respectively.*

**TABLE 2:** The initial panel of SL gene pairs relevant to oral cancer.

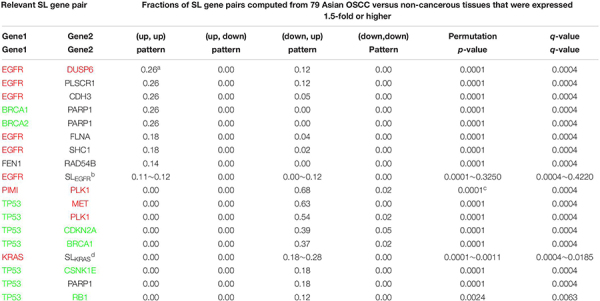

*^*a*^The four fractions were computed from gene pairs that were 1.5-fold differentially expressed, thus they might not sum up to 100%.*

*^*b*^Four verified EGFR SL pairs were identified in the (up, up) pattern.*

*^*c*^The p-value shows the significance of the (down, up) pattern.*

*^*d*^26 verified KRAS SL pairs were identified in the (down, up) pattern.*

*The *p*-value for the highest fraction four patterns was computed by permutation test with 10,000 repeats, and the false discovery rate was estimated by *q*-value.*

*Fractions of the four differentially expressed patterns based on the 1.5-fold threshold and filtered from 742 synthetic lethal gene pairs.*

### Immunohistochemistry Staining

Patients cancer samples were cut into 4-μm-thick sections and deparaffinized in xylene as previously described (Chang et al., 2016). Endogenous peroxidase activity was quenched with 3% (v/v) H_2_O_2_. The sections were boiled in 10 mM citrate buffer for 20 min to revive the antigens. The tissues were incubated with 21 primary antibodies at room temperature for 30 min then rinsed three times with phosphate-buffered saline (PBS) (Supplementary Table 1) according to the manufacturer’s protocol. The tissues were then incubated at 25°C for 30 min with secondary antibodies and a horseradish peroxidase/Fab polymer conjugate [EnVision detection systems peroxidase/DAB, rabbit/mouse (K5007 HRP; DaKo)] then rinsed three times with PBS. Finally, chromogen was developed using 3,3′-diaminobenzidine tetrahydrochloride as the substrate, and counterstained with hematoxylin and viewed under a microscope. Staining intensity in the cancer tissue was independently examined by two pathologists (Chun-Chieh Wu and Yi-Ting Chen).

The scoring criteria used here were the same as those of previous studies (Su et al., 2004; Tiong et al., 2014; Chang et al., 2016) (Supplementary Table 2). Stain intensity is graded as negative (0), indeterminate (±), weakly positive (1+), moderately positive (2+), or strongly positive (3+). The criterion is exactly based on the strongest intensity followed by the % expression of the detected protein. Negative (0) indicates no expression of the detected protein, indeterminate means that the staining is weak and its percentage cannot be accurately counted, weakly positive indicates <5% expression of the detected protein, moderately positive is focal expression in 5–20% of the cancer cells, and strongly positive indicates diffuse expression in >20% of the cancer cells. The mean staining intensity of three cancerous tissues was compared with that of noncancerous oral mucosa and was categorized as either over- or underexpressing, determining the criteria for IHC analyses. The cutoffs for the 29 IHC stains are listed in Table 3.

**TABLE 3:** Immunohistochemistry (IHC) proteins derived from cancerous tissues which were sampled from 163 local oral cancer patients, the cutoff values for over- and under-expression of IHC.

No.	Protein	Criterion for	Criterion for
No.	name	under-expression	over- expression
1	BRCA1(N)^a^	<1+	≧1+
2	CDH3(C)^a^	<1+	≧1+
3	CDH3(N)	<1+	≧1+
4	CDK6(C)	≤1+	>1+
5	CSNK1E(C)	≤1+	>1+
6	EGFR(C)	≤1+	>1+
7	EGFR(M)^a^	<1+ and < 10%^b^	≧1+ and ≧10%^b^
8	FEN1(C)	<1+	≧1+
9	FLNA(C)	<1+	≧1+
10	FLNA(N)	≤1+	>1+
11	KRAS(C)	<1+	≧1+
12	MET (C)^c^	≤1+	>1+
13	MSH2(N)	<1+	≧1+
14	P16(C)	≤1+	>1+
15	P16(N)	<1+	≧1+
16	PARP1(N)	<1+	≧1+
17	PIM1(C)	<3+	≧3+
18	PIM1(N)	<3+	≧3+
19	PLK1(C)	<3+	≧3+
20	POLB(C)	≤ 1+	>1+
21	POLB(N)	≤ 1+	>1+
22	RAD54B(N)	<1+	≧1+
23	RB1(N)	<1+	≧1+
24	SGK2(C)	≤1+	>1+
25	SHC1(C)	<1+	≧1+
26	SHC1(N)	≤1+	>1+
27	STK17A(C)	≤1+	>1+
28	STK17A(N)	≤1+	>1+
29	TP53(N)	≤0%	>0%

*^*a*^The notation (C), (N) and (M) represent cytoplasm, nucleus and membrane, respectively.*

*^*b*^Both strength of staining ≥ 1+ and stained area ≥ 10% are required.*

*^*c*^Phosphorylated MET was stained.*

*^*d*^The stained area > 0% is required.*

### Log-Rank Test

For each individual and paired IHC staining, a log-rank test of the “high-” and “low-” risk patients was conducted. The high- and low-risk groups consisted of patients classified according to the IHC amounts shown in Table 3. If the log-rank test was significant (*P* < 0.05), which indicated the survival curves of the two groups significantly different, then a Kaplan-Meier survival curve was plotted by the R software.

### Univariate Cox Proportional Hazard (PH) Regression Models

In the univariate Cox regression models, the associations between the 29 individual IHC or 398 combined IHC staining pairs and the 10-year overall survival of the OSCC patients were analyzed in the study cohort. The associations between clinical factors such as age (>55 vs. ≤55 years), sex (male vs. female), tumor grade (medium and high vs. low), lymph node metastasis (yes vs. no), stage (III, VI vs. I, II), and habits alcohol use (yes/no), betel nut chewing (yes/no), and cigarette smoking (yes/no) with 10-year Taiwanese OSCC overall survival were also assessed.

### Multivariate Cox PH Regression Model

When fitting the multivariate Cox regression models, the clinical factor stage significantly associated with overall survival in the univariate Cox regression models was adjusted, because the stage had stronger significance than that of the grade. Likelihood ratio test (*LR*_χ^2^_) and the statistical significance values generated (*P* values) were used to compare model fit between the uncovered prognostic IHC markers.

### Determination of the Cutoff for Differential Expression of the TCGA Data

We first used 1.5-fold as the threshold for differential TCGA OSCC gene expression, but there were too few patients (less than 5) (Vittinghoff and McCulloch, 2007) in the poor/good overall survival subsets to perform univariate Cox regression for most of the six prognostic markers (Table 4A). Thus, the cutoff was relaxed to 1.4-fold, and there were ensure adequate numbers of patients in the poor/good overall survival subsets of two pairs *CDH3-STK17A* and *FLNA-KRAS*, respectively, for the univariate Cox regression analysis.

**TABLE 4:** Overall survival of 153 oral squamous cell carcinoma patients relative to clinical covariates, IHC prognostic markers, and habits.

**A. Univariate Cox regression**				
**Variable**	**Subset**	**Hazard ratio (95% CI)**	***p*-value**	***LR*_x^2^_**
Lymph node metastasis	yes/no	3.47 (1.92–6.28)	0.000	15.6
Stage	III–IV/I–II	3.15 (1.67–5.95)	0.000	13.1
Grade	low, moderate and high	1.94 (1.06–3.54)	0.031	4.8
RB1(N)	↑/↓^a^	2.03 (1.07–3.86)	0.031	4.7
[CSNK1E(C), SHC1(N)]	(↓, ↓)/otherwise	7.54 (3.08–18.43)	0.000	12.8
[CSNK1E(C), RB1(N)]	(↓, ↑)/otherwise	2.92 (1.46–5.83)	0.002	7.6
[CDH3(C), STK17A(N)]	(↑, ↑)/otherwise	3.58 (1.27–10.10)	0.016	5.4
[BRCA1(N), SHC1(N)]	(↓, ↓)/otherwise	2.96 (1.15–7.59)	0.024	4.2
[FLNA(C), KRAS(C)]	(↑, ↑)/otherwise	0.49 (0.25–0.96)	0.039	3.9

**Habit**	**Subset**	**Hazard ratio (95% CI)**	***p*-value**	***LR*_x^2^_**

Alcohol use	Yes/No	2.01 (1.01–3.97)	0.045	4.4

**B. Multivariate Cox Regression^b^**				

**Variable**	**Subset**	**Hazard ratio (95% CI)**	***p*-value**	***LR*_x^2^_**

RB1(N)	↑/↓	1.71(0.89–3.30)	0.108	16.2
Stage	III–IV/I–II	3.18(1.65–6.14)	0.001	
[CSNK1E(C), SHC1(N)]	(↓, ↓)/otherwise	7.75(2.85–21.07)	5.9 × 10^–5^	23.3
Stage	III–IV/I–II	3.45(1.74–6.85)	4.1 × 10^–4^	
[CSNK1E(C), RB1(N)]	(↓, ↑)/otherwise	2.16(1.02–4.58)	0.045	16.4
Stage	III–IV/I–II	3.04(1.57–5.87)	0.001	
[BRCA1(N), SHC1(N)]	(↓, ↓)/otherwise	2.87(1.11–7.42)	0.030	17.1
Stage	III–IV/I–II	3.34 (1.68–6.61)	0.001	

**C. Combination of two gene pairs**				

**Variable**		**Hazard ratio (95% CI)**	***p*-value**	***LR*_x^2^_**

CSNK1E(C)-SHC1(N) (↓, ↓) and FLNA(C)-KRAS(C) (↑, ↑)^c^*		8.71(2.88–26.36)	0.000	1.98
Stage		2.95(1.45–6.02)	0.003	
BRCA1(N)-SHC1(N) (↓, ↓) and FLNA(C)-KRAS(C) (↑, ↑)		3.14 (1.2–8.24)	0.020	14.94
Stage		2.91 (1.42–5.95)	0.004	

*^*a*^The symbols “↑” and “↓” denote over- and under-expression of IHC, respectively of the corresponding protein.*

^*b*^Variables were selected by stepwise selection and AIC.

*The symbol (↑, ↑);^*c*^denotes the complementary set of (↑, ↑), namely (↓, ↑), (↑, ↓) and (↓, ↓), in which FLNA(C)-KRAS(C) is in the same direction (poor OS) as that of BRCA1(N)-SHC1(N).

## Results

### Description of Study Population

As shown in Table 1, about half of the patients in our study cohort were <55 years old at the time of diagnosis. The histologic grades were defined as low grade: well differentiated, intermediate grade: moderately differentiated, and high grade: poorly differentiated. Most of the cancers (98%) were intermediate- or low histological grade, only 1.3% were high grade. About 60% of the patients were stage I and II, and 26.5% were stage IV (most of them were stage IVA). All cancers were squamous cell carcinoma. According to the stratification of 7th version of the American Joint Committee on Cancer (AJCC), 71.2% of the tumor sizes belonged to T1 and T2, and 17.2% belonged to T4. Most of the lymph node statuses were N0 and N1 (85.3%).

### Initial Panel of Relevant SL Gene Pairs for OSCC

In general, tumor cells show aberrant expression of oncogenes and tumor suppressor genes. Validated SL pairs comprised oncogenes and tumor suppressor- and genome stability genes. Therefore, we first selected gene pairs relevant to OSCC from the 742 SL pairs, using the microarray gene expression data of 57 Asian OSCC and 22 non-cancerous tissues {GSE 25099 from the gene expression omnibus database [GEO (Srivastava and Raghavan, 2015)]} (Peng et al., 2011). The selected SL gene pairs were further sorted by the fractions of the (up, up), (down, up), (up, down), and (down, down) patterns (Table 2), where up and down denoted upregulation and downregulation with the cutoff 1.5-fold; this less stringent cutoff was set to include important OSCC onco- and tumor suppressor genes not expressed at twofold level, e.g., *TP53*, *EFGR*, and *CDKN2A*, but that were frequently mutated in Asian OSCC (Liu et al., 2004; Presson et al., 2011). Overexpression of tumor suppressor- and genome stability gene pairs associated with DNA repair such as *BRCA1* and *FEN1* was unexpectedly noted (Table 2). However, this finding was consistent with the dramatic increase in genomic instability and DNA replication caused by mutant oncogenes such as *MYC*.

### Twenty-One Genes Were Selected for IHC Staining

We selected 21 genes from Table 2 to conduct IHC staining, and some of them were stained at two cellular portions. Most of the genes were selected according to relatively high fractions of the (up, up) and (down, up) patterns (≥15%) in Table 2. For an extended list of the sorted (up, up) and (down, up) gene pairs, please see^2^. Next, we applied prior knowledge to (i) select *CDH3* from the *EGFR* SL pairs and *STK17A* and *CDK6* from the *KRAS* SL pairs, which satisfied the above fraction cutoffs, and to (ii) include genes whose fractions were on the borderline of 15%; this included *FEN1-RAD54B* (Srivastava and Raghavan, 2015) (14%), *RB1* (Liu et al., 2004; Presson et al., 2011) (12%) and *MSH2-POLB* (Kang et al., 2009; Tiong et al., 2014; Chang et al., 2016). Please see the section “Materials and Methods” for details of the selection method.

**FIGURE 2:**
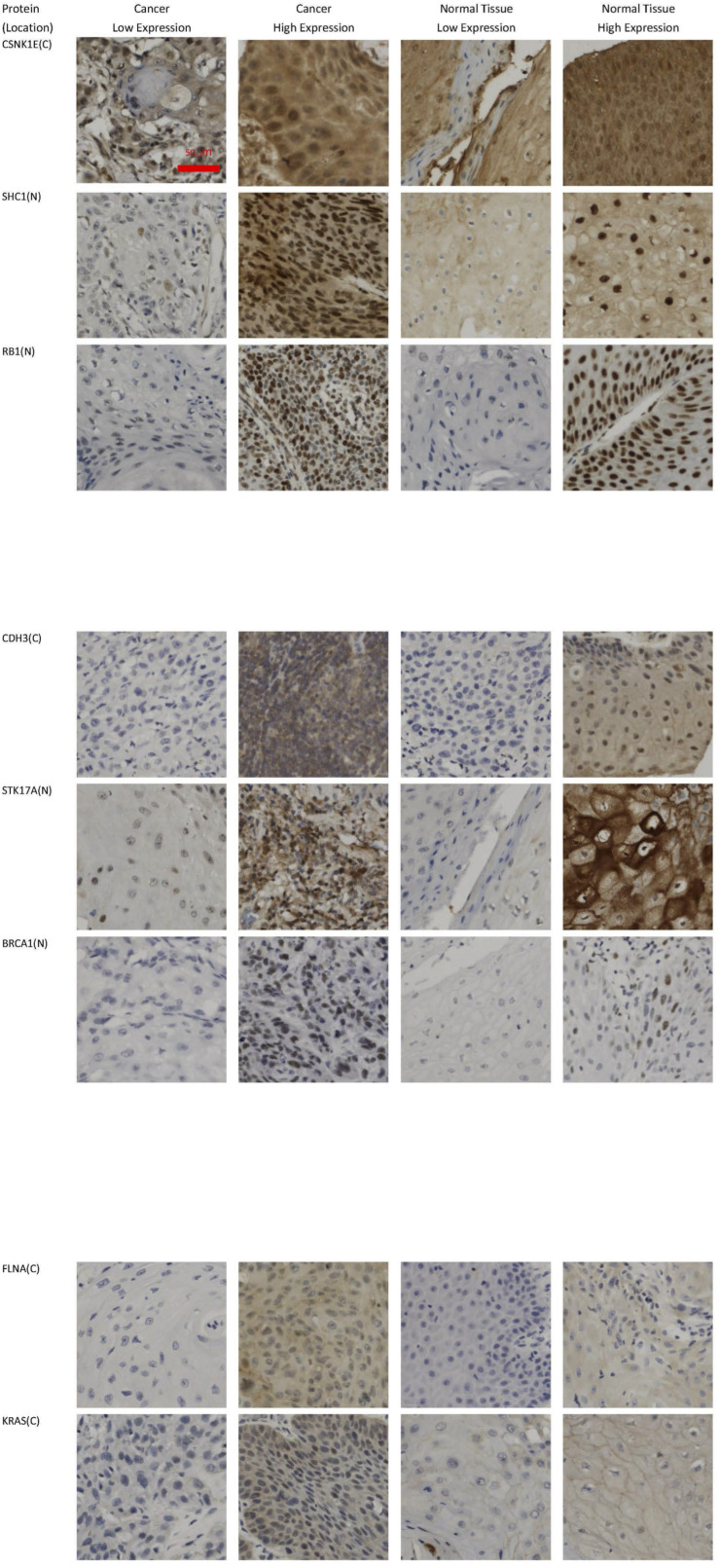
Representative IHC images. Over- and underexpression of IHCs involved in the revealed markers [CSNK1E(C), SHC1(N), RB1(N), CDH3(C), STK17A(N), BRCA(N), FLNA(C), and KRAS(C)] are shown for cancer and normal tissues from OSCC patients (original magnification: × 400).

Eight out of these 21 genes were stained at two cellular portions, such as *CDH3* and *EGFR*, the remaining 13 genes were stained at one cellular portion. Table 3 lists these 29 different IHC stains, the cutoffs for over- and underexpression of IHC staining and the corresponding fractions of OSCC patients satisfying the cutoffs. See section “Materials and Methods” for the basis determining the cutoff values. Some representative IHC figures are shown in Figure 2, including CSNK1E(C), SHC1(N), RB1(N), CDH3(C), STK17A(N), BRCA(N), FLNA(C), and KRAS(C). The IHC figures of all proteins are in Supplementary Figure 1.

**FIGURE 3:**
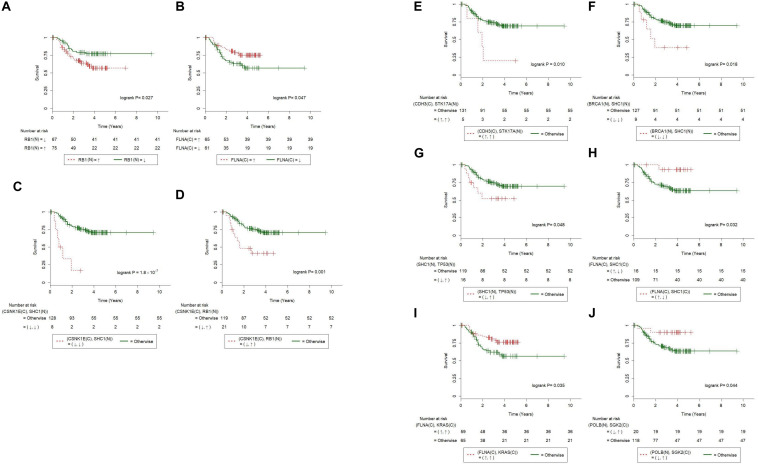
Immunohistochemistry of individual and paired proteins correlated with overall survival of 153 Taiwanese oral squamous cell carcinoma patients. Kaplan-Meier survival curves were significantly different in terms of **(A)** RB1(N), **(B)** FLNA(C), **(C)** CSNK1E(C)-SHC1(N), **(D)** CSNK1E(C)-RB1(N), **(E)** CDH3(C)-STK17A(N), **(F)** BRCA1(N)-SHC1(N), **(G)** SHC1(N)-TP53(N), **(H)** FLNA(C)-SHC1(C), **(I)** FLNA(C)-KRAS(C), and **(J)** POLB(N)-SGK2(C). Curves for patients with paired abnormal IHCs (according to Table 3) are plotted with dashed lines. Curves for the other patients are plotted with solid lines. The symbols ↑ and ↓ denote overexpression and underexpression of the corresponding IHCs, respectively.

We next explored if the results of IHC stains are suitable for use as prognostic markers. For each of the 29 IHC results in Table 3 and all of the combined IHC pairs, we applied log-rank tests to the 153 Taiwanese patients with OSCC for whom overall survival was recorded. We first observed that the patients with overexpressed RB in nucleus (denoted as RB1(N)↑) had significantly poorer overall survival than patients with underexpressed RB1 (*P* = 0.027, Figure 3A). Additionally, underexpressed FLNA in cytoplasm [FLNA(C)↓] was also associated with poor clinical outcomes (*P* = 0.047, Figure 3B).

## RB1↑ and FLNA(C)↓ Were Associated With Poor Overall Survival

### IHC of Eight Protein Pairs Were Associated With Overall Survival

Furthermore, we combined the 29 IHC stains into all the possible distinct IHC pairs (398 in total), which allowed novel paired IHC markers to be uncovered, excluding those of the same protein stained at different cellular portions. Univariate Cox regression procedure revealed that CSNK1E(C)↓-SHC1(N)↓, CSNK1E(C)↓-RB1(N)↑, CDH3(C)↑-STK17A(N)↑, BRCA1(N)↓-SHC1(N)↓, and SHC1(N)↓-TP53↑ were associated with poorer overall survival (Figures 3C–G; P = *1.8×10^−7^*, 0.001, 0.010, 0.018, and 0.048, respectively; log-rank test). On the other hand, FLNA(C)↑-SHC1(N)↓, FLNA(C)↑-KRAS↑, and POLB↓-SGK2↑ were correlated with better overall survival (Figures 3H–J; P = 0.032, 0.035, and 0.044, respectively; log-rank test).

### Multivariate Cox Regression Analysis Revealed That CSNK1E↓-SHC1(N)↓, CSNK1E↓-RB1↑, and BRCA1(N)↓-SHC1(N)↓Were Independent Prognostic Markers

As reported previously, biomarkers can be identified from gene- or protein expression data (Presson et al., 2011; Ha et al., 2015). For the 29 IHC results, univariate Cox regression models (Table 4A) confirmed that RB1(N) [hazard ratio (95% confidence interval) = 2.03 (1.07–3.86); *P* < 0.05] was a prognostic marker. The univariate Cox regression analysis was also applied to the combined IHC pairs. The results suggested that CSNK1E↓-SHC1(N)↓ [hazard ratio (95% confidence interval) = 7.54 (3.08–18.43); *P* < 0.001], CSNK1E↓-RB1↑ [hazard ratio (95% confidence interval) = 2.92 (1.46–5.83); *P* = 0.002], CDH3(C)↑-STK17A(N)↑ [hazard ratio (95% confidence interval) = 3.58 (1.27–10.10); *P* = 0.016], BRCA1(N)↓-SHC1(N)↓ [hazard ratio (95% confidence interval) = 2.96 (1.15–7.59); *P* = 0.024], and FLNA(C)↑-KRAS↑[hazard ratio (95% confidence interval) = 0.49 (0.25–0.96); *P* = 0.039] were significant predictors of the risk of death in Asian patients with OSCC (Table 4). In addition, the paired markers CSNK1E↓-SHC1(N)↓ (*LR${}_{x}^{2}$* = 12.8) and CSNK1E(C)↓-RB1(N)↑ (*LR${}_{x}^{2}$* = 7.6) provided more powerful prognostic information than the individual marker RB1(N) (*LR${}_{x}^{2}$* = 4.7). There were too few patients in the MSH2↓-TP53↑ and MSH2↓-SHC1↓ subsets to perform univariate Cox regression analysis.

Of the clinical variables [age, sex, tumor grade, lymph node (LN) metastasis and stage], grade, lymph node metastasis and stage were significantly associated with the patients’ overall survival (Table 4A). The univariate model based on stage (*LR${}_{x}^{2}$* = 13.1) fit better than that based on grade (*LR${}_{x}^{2}$* = 4.8). Therefore, we used stage as the adjustment factor in the multivariate Cox regression models.

Because the high incidence of oral cancer in Asian OSCC is related to alcohol use, betel nut chewing, and cigarette smoking, we investigated whether these habits were associated with overall survival in this population. As shown in Table 4A, only alcohol use [hazard ratio (95% confidence interval) = 2.01 (1.01–3.97); *P* = 0.045] was a significant predictor of overall survival in these Taiwanese patients with OSCC. Betel nut chewing [hazard ratio (95% confidence interval) = 0.72 (0.38–1.38); *P* = 0.329] and smoking [hazard ratio (95% confidence interval) = 1.79 (0.64–5.00); *P* = 0.267] were not significant predictors for the risk of death in these patients. Furthermore, the correlation between alcohol use and each IHC marker was tested (Fisher’s exact test) and none was significant at *P* = 0.05. Similarly, the correlation between “the combined habits” and each IHC marker was tested, and again none was significant at *P* = 0.05; please see Supplementary Table 3 for details.

We then evaluated the associations of the novel IHC prognostic markers with overall survival after adjusting for alcohol use, age and tumor stage, *via* multivariate Cox regression analysis. The paired prognostic markers CSNK1E↓-SHC1(N)↓ [hazard ratio (95% confidence interval) = 7.75 (2.85–21.07); *P* = 5.9 × 10^–5^], CSNK1E↓-RB1(N)↑ [hazard ratio (95% confidence interval) = 2.16 (1.02–4.58); *P* = 0.045], and BRCA1(N)↓-SHC1(N)↓ [hazard ratio (95% confidence interval) = 2.87 (1.11–7.42); *P* = 0.030] were significant predictors of the overall survival of the patients with OSCC. For patients with CSNK1E↓-SHC1(N)↓, CSNK1E↓-RB1(N)↑, and BRCA1(N)↓-SHC1(N)↓, the risk of death was 7.8, 2.2, and 2.9 times higher, respectively, than that for the other patients in this population. However, RB1(N) [hazard ratio (95% confidence interval) = 1.71 (0.89–3.30); *P* = 0.108] was no longer a significant predictor (Table 4B). After alcohol use, age, and stage were entered into the multivariate Cox regression models along with each of the markers, neither alcohol use nor age was selected by stepwise selection or Akaike information criterion (AIC). Thus, neither appeared in the final models (Table 4B). Following a reviewer’s suggestion, we further adjusted the effect of lymph node metastasis and tumor stage in the multivariate Cox regression models, because lymph node density and metastasis were shown to be significant prognosis predictors in OSCC (Zanaruddin et al., 2013; Chang et al., 2018). Excluding the effect of LN metastasis and stage that are used in clinical practice conventionally, the revealed five combined markers are still significant (Supplementary Table 4). This highlights the potential of these markers being targeted for cancer treatments.

### Combinations of Significant Markers Were Studied; CSNK1E↓-SHC1(N) Was Suggested for Clinical Practice

We then combined any two of the significant markers in Table 4A and selected eight combinations whose good/poor OS subsets consisted of a sufficient number of (≥5) patients. Note that the (↑,↑) subset of FLNA(C)-KRAS(C) was correlated with a good OS, so we combined its complementary subsets (↓, ↑), (↑, ↓), and (↓, ↓) with the poor OS subsets of the remaining five markers in Table 4A. We fitted multivariate Cox regression to these combinations, and found that [CSNK1E-SHC1(N), FLNA(C)-KRAS(C)] (hazard ratio = 8.71; *P* = 0.000 rounded to three decimal places) and [BRCA1(N)-SHC1(N), FLNA(C)-KRAS(C)] (hazard ratio = 3.14; *P* = 0.020) were significant prognostic markers (Table 4C). For combinations of three or more significant markers, the (good/poor OS) subsets had too few patients to fit any multivariate Cox regression model. Taking Table 4A–C together, we suggest using the combination CSNK1E↓-SHC1(N)↓, which has the most significant *P* value (from likelihood-ratio test) among all markers, to identify Asian OSCC patients with worst survival in clinical practice.

### External Validation of the Association of CDH3-STK17A With Overall Survival

Ethnicity and geography may play a role in the etiology of cancer. If the newly discovered markers are confirmed by independent datasets of patients with OSCC from different geographic regions and ethnicities, they may be useful tools in clinical medicine. OSCC with HPV(−) from the TCGA head and neck SCC cohort (henceforth, TCGA) (The Cancer Genome Atlas Network, 2015) more closely resembled Asian OSCC than those with HPV(+). Therefore, we analyzed microarray gene expression data of 160 OSCC cases with HPV (−) to validate the novel prognostic markers in Table 4.

**FIGURE 4:**
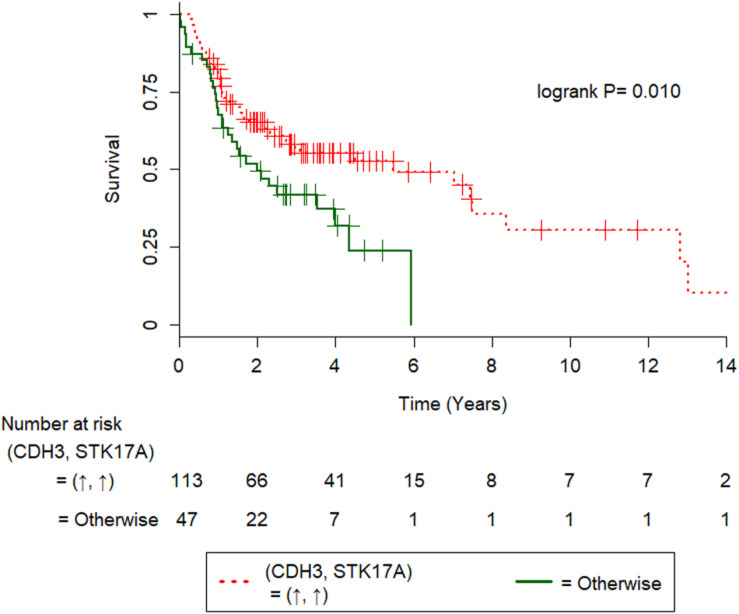
Kaplan-Meier survival curves of 160 HPV(−) oral squamous cell carcinoma patients from the TCGA cohort. Kaplan-Meier survival curves were significantly different in terms of gene expression for CDH3-STK17A, where the symbols ↑ and ↓ denote overexpression and underexpression of the corresponding genes at the 1.4-fold cutoff.

We used 1.4-fold as the cutoff for differential expression of TCGA OSCC RNA-seq data (see section “Materials and Methods” for details), such that of all markers in Table 4A, *CDH3-STK17A* and *FLNA-KRAS* had a sufficient number of (five or more) (Vittinghoff and McCulloch, 2007) patients in the (good/poor OS) subsets for the univariate Cox regression analysis. Of these, *CDH3↑-STK17A↑* was a significant gene predictor of good survival [hazard ratio (95% confidence interval) = 0.55(0.35–0.87); *P* = 0.011], while *FLNA↑*-*KRAS↑* was not significant (*P* = 0.117). The former finding was not consistent with ours, which showed that CDH3(C)*↑-*STK17A(N)*↑* was a significant predictor of poorer overall survival in Taiwanese patients with OSCC (Table 4A). This discrepancy may be explained by the different genetic backgrounds in the two populations, since the significant downregulation of the *CDH3* gene has been reported in metastatic OSCC (Méndez et al., 2009). The estimated survival curves of *CDH3-STK17A* are shown in Figure 4. This external validation demonstrates that if IHC or gene expression data are available, CDH3-STK17A can be used to stratify patients with OSCC in the future.

## Discussion

Here, we established a cost-effective approach for the identification of prognostic IHC markers of OSCC. This approach is also efficient, as merely 29 IHC stains were performed, but five clinically beneficial prognostic markers were identified through extensive statistical analysis. Our technique rapidly uncovered the prognostic markers without any prerequisite knowledge of the molecular pathways. In contrast, previous studies relied on pathway information (Sadanandam et al., 2013; Kosari et al., 2014) to reveal prognostic markers, such as cellular phenotypes and protein expression levels. Moreover, our approach was able to reveal IHC prognostic markers with components from different pathways. This improves the current state of art, as most of methods to uncover IHC markers to date have been mainly based on one or two proteins (Lin et al., 2014), or one pathway (Oliveira-Costa et al., 2014).

Of the single IHC results, RB1(N) was a predictor of poorer survival in the Taiwanese patients with OSCC, however, it was not independent of tumor stage. This finding was consistent with earlier studies wherein RB1 was a biomarker in HPV(−) head and neck cancers (The Cancer Genome Atlas Network, 2015; Beck et al., 2016). Previous studies showed that expression of Rb increased in the development and/or with disease progression of OSCC (Pavelic et al., 1996; Schoelch et al., 1999; Thomas et al., 2015), and the latter study reported high expession of Rb in patients with combined habits (alcohol use, betel nut chewing and smoking), suggesting Rb pathway altered. However, in our study, the over-expression of Rb was confounded with stage (Tables 4A,B), but not associated with the combined habits (*P* = 0.19, Fisher’s exact test; Supplementary Table 3). The over-expression of RB1(N) in our study may be due to over-expression of cyclin D1 or under-expression of p16^*INK4A*^, as cyclin D1 and p16^*INK4A*^ are related to Rb through an autoregulatory loop (Gimenez-Conti et al., 1996; Andl et al., 1998). Although expression of RB1(N) was high in our study, its function was likely inactivated which may be due to regulation of cyclin D1, HPV infection (Gimenez-Conti et al., 1996; Andl et al., 1998), loss of heterozygosity (Maestro et al., 1996; Yokoyama et al., 1996) or Rb hyperphosporylation (Chatterjee et al., 2004), but further studies are required to elucidate this.

Of the 398 IHC pairs, multivariate Cox regression analyses showed that CSNK1E↓-SHC1(N)↓, CSNK1E↓-RB1(N)↑, and BRCA1(N)↓-SHC1(N)↓ were significant predictors of the risk of death in this Taiwanese OSCC population, independent of tumor stage. Of all combinations of two significant markers in Table 4A, [CSNK1E-SHC1(N), FLNA(C)-KRAS(C)] was the most significant poor prognostic factor. Nevertheless, this marker was less significant than CSNK1E↓-SHC1(N)↓ statistically. Thus, in clinical practice we recommend using CSNK1E↓-SHC1(N)↓ to identify patients with severe and/or advanced Asian OSCC, who should be suggested for alternate or more intense treatment strategies in clinical practice. CK1 ϵ could be an oncoprotein or a tumor suppressor (Lin et al., 2014), but phosphorylation of CK1 ϵ can stabilize and activate tumor suppressor p53 (Knippschild et al., 2005). SHC1 is a known downstream target of p53, which involves in stress-induced signal transduction pathway (Trinei et al., 2002). Moreover, SHC1 was downregulated by miR-5582-5p, thus led a tumor suppressive activity with GAB1 (An et al., 2016). In our study, the mean survival rate of OSCC patients with CSNK1E↓-SHC1↓ is 13.8 months compared to 37.8 months of the remaining group. Collectively, CSNK1E-SHC1 might be a tumor suppressor, but this requires further studies for elucidation. As phosphorylation of CK1 ϵ can stabilize and activate tumor suppressor p53, moreover, expression of p53 was lower in OSCC lesions than in malignant lesions, and Rb expression was observed in OSCC lesions (Oliveira and Ribeiro-Silva, 2011). Thus, we speculate p53 may indirectly interfere with RB1, after p53 been regulated by phosphorylated CK1, which supports the finding CSNK1E↓-RB1(N)↑ is a poor prognostic marker.

External gene expression data of HPV(−) OSCC from the TCGA cohort (98.7% non-Asian patients) validated that *CDH3↑-STK17A↑*as a significant predictor of good survival in 160 patients with HPV(−) OSCC, where the cutoff was set at 1.4-fold. Nevertheless, when we set the cutoff at 1.5-fold, this gene pair was no longer significant (*P* = 0.124). Thus, this gene pair may not be a robust prognosis marker. Our result showed that CDH3(C)↑-STK17A(N)↑ was correlated with poor survival of 163 Taiwanese patients with OSCC. The difference in the aforementioned results may be because overexpression of P-cadherin (coded by CDH3) in membrane was associated with good survival of 67 OSCC patients, however, cytoplasmic expression of P-cadherin was correlated with poor survival (Muzio et al., 2005). Furthermore, high cytoplasmic expression of STK17A was reported to increase tumorigenic potential through inhibition of TGF-beta1-mediated tumor suppressor activity in HNSCC cells (Park et al., 2015).

Some of the prognostic markers our approach revealed are well-known and reported in the literature, thus we performed a comparative analysis as follows. Among all biomarkers, CSNK1E*↓*-SHC1(N)*↓* was the most significant in terms of *P* < 0.0001 (Table 5). Consistently, the loss of CK1ε expression was shown to be a poor prognostic marker in Taiwanese patients with oral cancer (Lin et al., 2014). Next, overexpression of cyclin D1 and Rb and low expression of p16 was significantly associated with reduced disease-free survival in 348 Indian patients with OSCC (Jayasurya et al., 2005), consistent with our findings that the overexpression of cytoplasmic Rb was a poor prognostic marker in Taiwanese patients. However, Soni et al. (2005) reported that 105 Indian patients with OSCC with loss of Rb expression had poor prognosis. This discrepancy may be explained by a recent finding (Sanidas et al., 2019) that there are many different forms of active Rb, and they have distinct functional properties. Both Shiraki et al. (2005) and our team found that overexpression of p53 (EGFR) was not a significant prognostic marker in OSCC, but the former study revealed that p53-Cyclin D1-EGFR was significantly associated with poor overall survival (*P* = 0.019). Moreover, BRCA1 overexpression was shown to be associated with reduced overall survival of 150 Brazilian patients with OSCC (Oliveira-Costa et al., 2014), whereas we did not find prognostic significance of BRCA1 underexpression, but BRCA1(N) ↓-SHC1(N)↓ was an independent prognostic marker (*P* = 0.024; Table 4B). This discrepancy may be due to the different genetic backgrounds of the populations.

**TABLE 5:** A comparison of our prognostic markers to those reported in literature.

Previous studies^*a*^		This study	
	
IHC marker	Sample size/*P* value	IHC marker	Sample size/*P* value
Cyclin D↑-Rb↑-p16↓^Jayasurya^	348/0.002	CSNK1E↓- SHC1(N)↓	163/5.9 × 10^–5^
Rb↑	348/0.062	CSNK1E↓- Rb↑	163/0.002
Rb↓^Soni^	98/0.036	Rb↑	163/0.031
Rb↓-p53↑^Soni^	98/0.004		
CSNK1E↓^Lin^	195/0.024	CSNK1E↓	163/insignificant^b^
p53-Cyclin D1-EGFR^Shiraki^	140/0.0019		
EGFR	140/insignificant	EGFR↑	163/insignificant
p53	140/insignificant	p53↑	163/insignificant
BRCA1↑^Oliveira^*	150/0.030	BRCA1(N)↓	163/insignificant
		BRCA1(N)↓- SHC1(N)↓	163/0.024
P-cadherin↓^Muzio^	67/0.056	CDH3(C)↑-STK17A (N)↑	163/0.016
		CDH3(C)↑	163/insignificant

*^*a*^The first author’s last name was indicated in the upper right corner of each study’s first marker.*

^*b*^*P* value ≥ 0.05.

*BRCA1↑ was associated with disease-specific survival.

In conclusion, our study revealed that the combined evaluation of CSNK1E↓-SHC1(N)↓ in OSCC identified a group of patients with the poorest survival, who should be suggested to undergo alternate or more intense treatment strategies. CK1ε combined with SHC adaptor protein 1 emerged as the most promising IHC prognostic marker in Asian OSCC. Of the 398 combined IHC pairs, genes of ten pairs are known to be SL, out of which only FLNA-KRAS was revealed to be a good OS marker, but not independent of stage. Excluding the effect of tumor stage and LN metastasis (Zanaruddin et al., 2013; Chang et al., 2018) that are used in clinical practice conventionally, the revealed markers of our study are still significant (Supplementary Table 4). This highlights the potential of these markers being targeted for cancer treatments.

Despite that we conducted a large scale of IHC study, the present study is limited by the moderate sample size and no genomic data profiled. Further studies based on larger sample sizes of patients with OSCC and on DNA sequencing data will reveal whether the expression of the uncovered IHC markers are due to their mutations. With ready availability of gene expression and tissue array data and resources to match clinicopathological features in the public and commercial domains, our approach can immediately be applied to other types of cancers. Moreover, additional IHC stain of cyclin D1 will enable us to evaluate the prognostic significance of protein triplets such as cyclin D1-Rb-p16 and p53-cyclin D1-EGFR. This is interesting, as the component of our most significant marker SHC adaptor protein 1-CK1↑ is involved in the EGFR pathway and is SL to both TP53 and EGFR, respectively. Given that the triplet IHC cyclin D1-Rb-p16 is a promising marker, future studies will extend to the prognostic effect of triplets of IHC in OSCC.

## Data Availability Statement

Part of the datasets in this study can be found in online repositories. The names of the repository/repositories and accession number(s) can be found in the articles. However, the clinical data that support the findings of this study are available from Kaohsiung Medical University Hospital with restrictions applying to the availability of these data, which were used under license for the current study, are not publicly available. Data are however available from the authors upon reasonable request and with permission from Kaohsiung Medical University Hospital.

## Ethics Statement

This study involving human participants was approved by the Institutional Review Board and Ethics Committee of Kaohsiung Medical University Hospital (KMUHIRB-E(I)-20170034). The data were analyzed anonymously, and therefore, no informed consent was required. All methods were performed under approved guidelines and regulations.

## Author Contributions

GS conceived the study. H-CW, C-CW, and Y-TC did IHC staining. C-JC implemented the methods, wrote the algorithm, and performed data analysis. H-CW, J-GC, and GS interpreted the data. H-CW and GS wrote the manuscript; T-CL modified H-CW’s writing in an earlier version. GS and T-CL designed and supervised the study. All authors read and approved the final manuscript.

## Conflict of Interest

The authors declare that the research was conducted in the absence of any commercial or financial relationships that could be construed as a potential conflict of interest.

## Footnotes

1 http://www.stat.sinica.edu.tw/~gshieh/OC/SL_pairs.html

2 https://www.stat.sinica.edu.tw/gshieh/OC/UU-DU_list
