# Supplementary material for: Immunohistochemical Expression of Five Protein Combinations Revealed as Prognostic Markers in Asian Oral Cancer
Source: Front Genet. 2021 Apr 15;12:643461. doi: 10.3389/fgene.2021.643461 (PMC8083901; doi:10.3389/fgene.2021.643461)
Supplement: Supplementary file 1 [file Image_1.pdf]

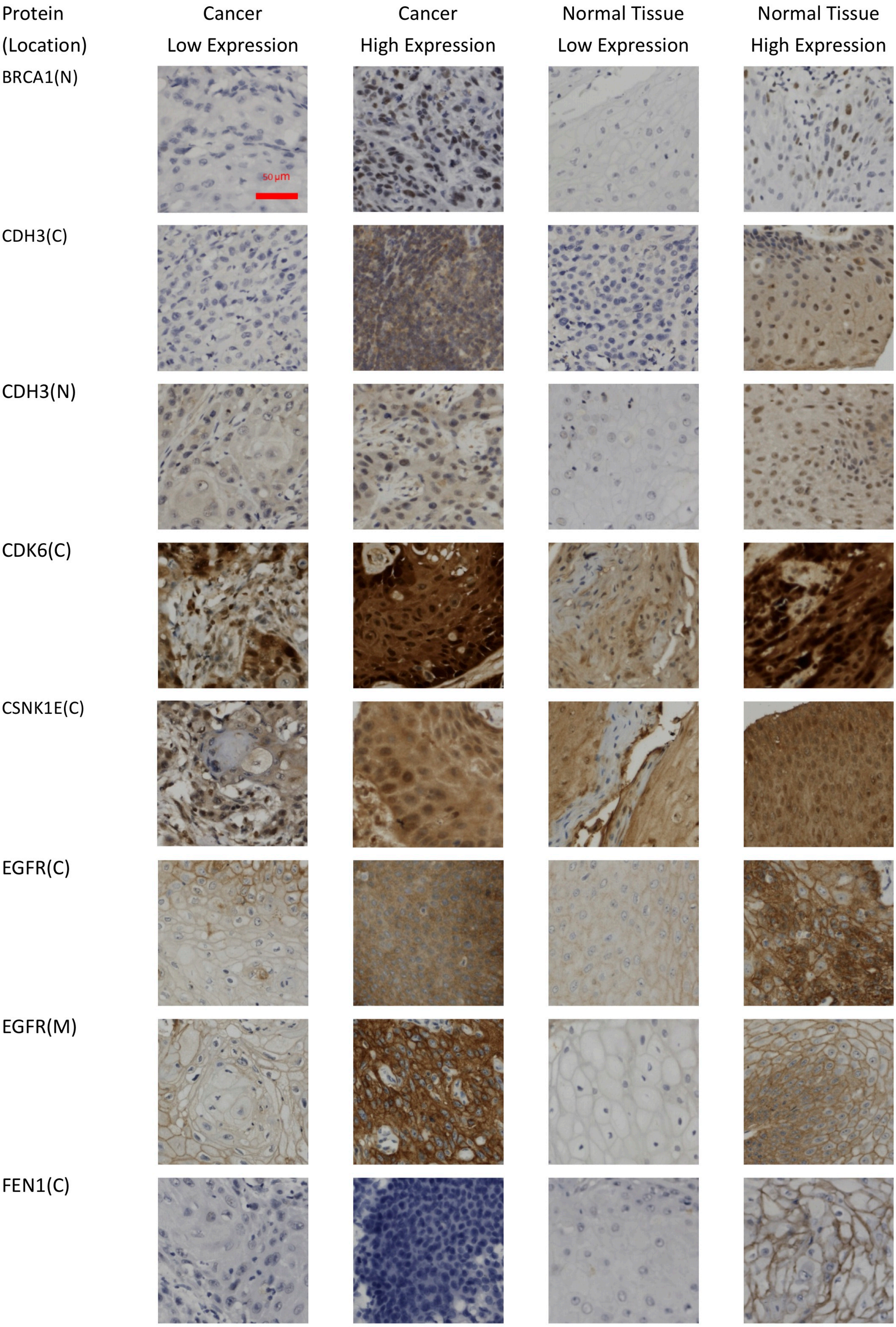
Protein
(Location)
Cancer
Low Expression
Cancer
High Expression
Normal Tissue
Low Expression
Normal Tissue
High Expression
BRCA1(N)
50 μm
CDH3(C)
CDH3(N)
CDK6(C)
CSNK1E(C)
EGFR(C)
EGFR(M)
FEN1(C)

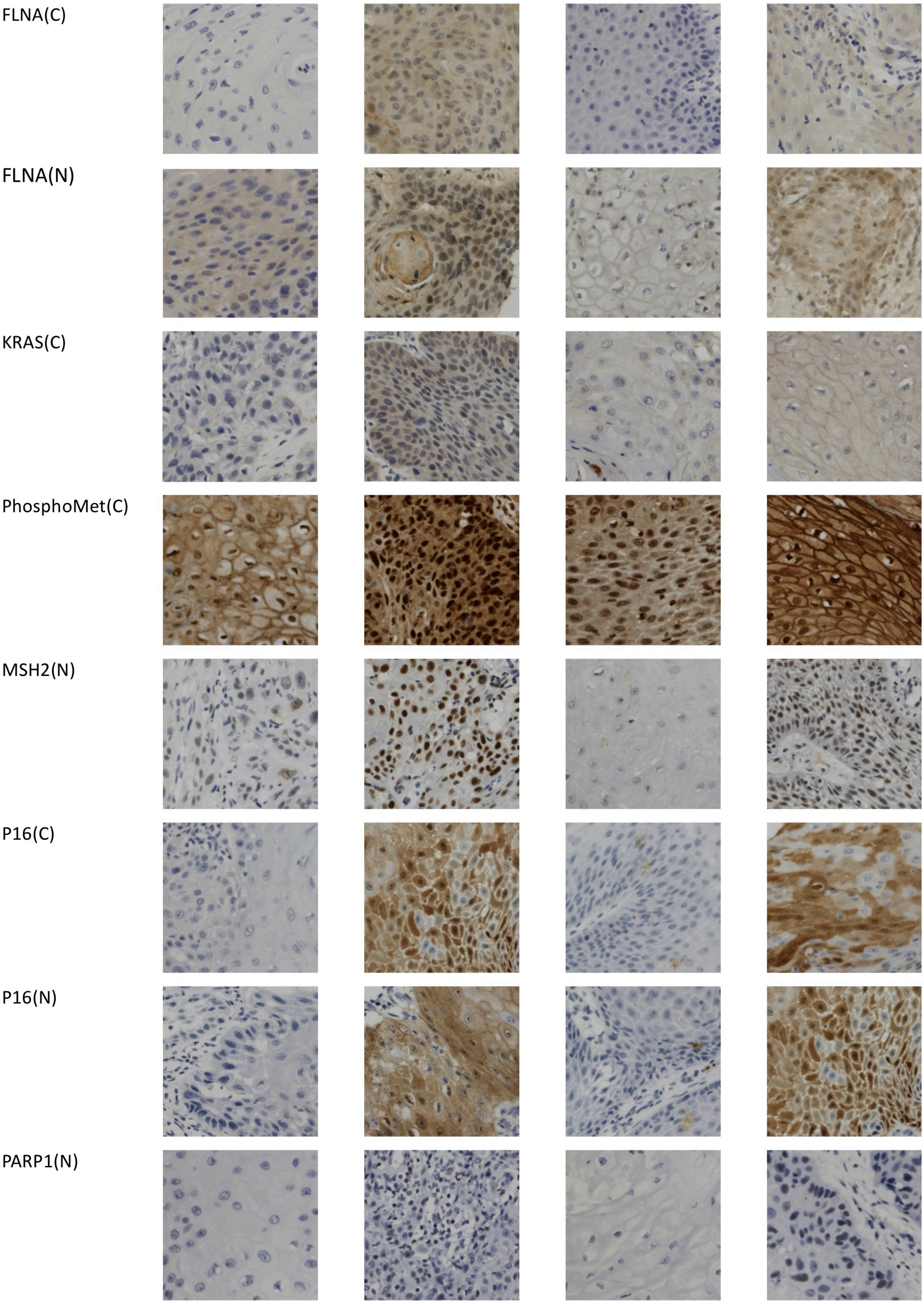
FLNA(C)
FLNA(N)
KRAS(C)
PhosphoMet(C)
MSH2(N)
P16(C)
P16(N)
PARP1(N)

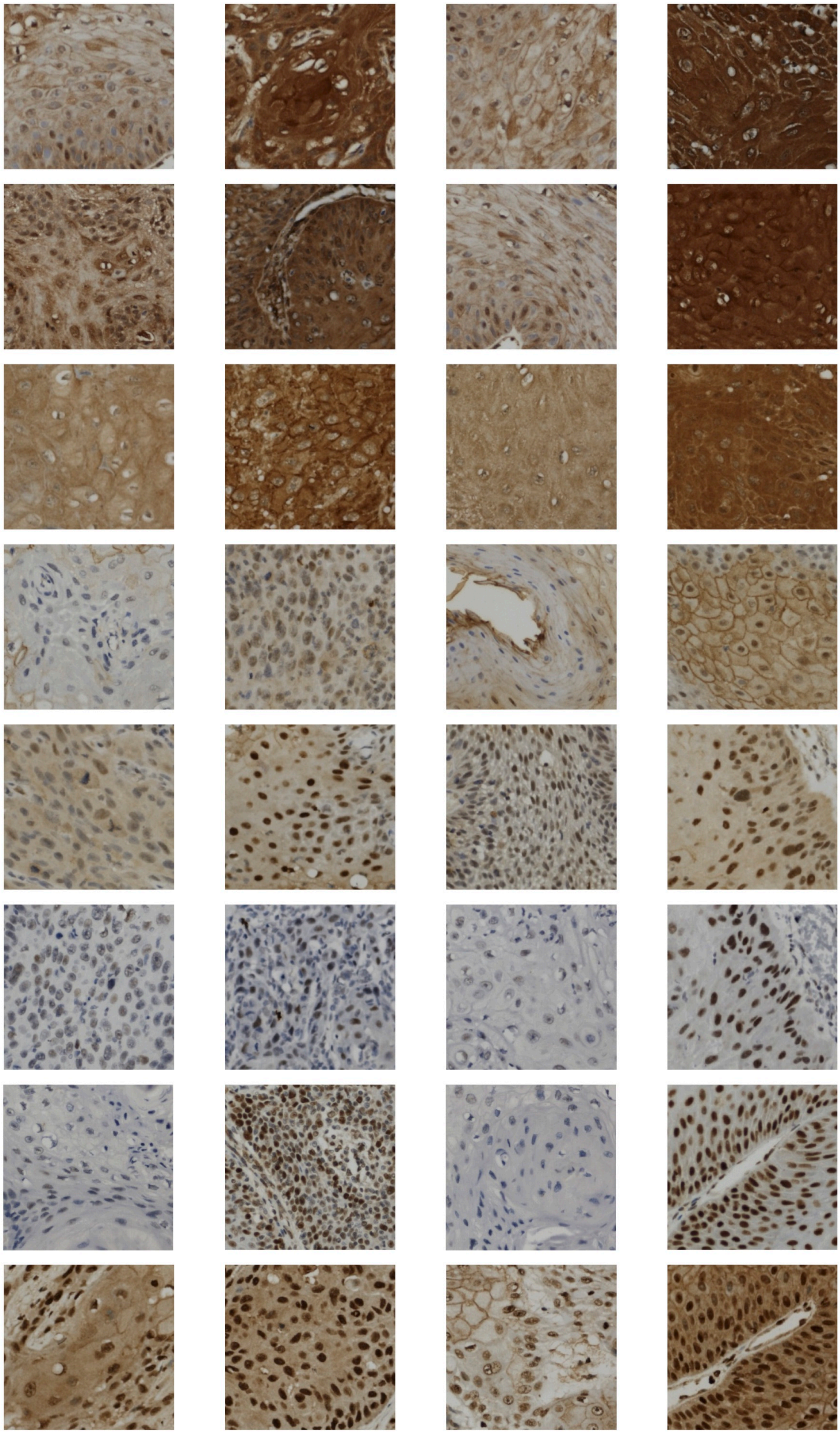

PIM1(C)
PIM1(N)
PLK1(C)
POLB(C)
POLB(N)
RAD54B(N)
RB1(N)
SGK2(C)

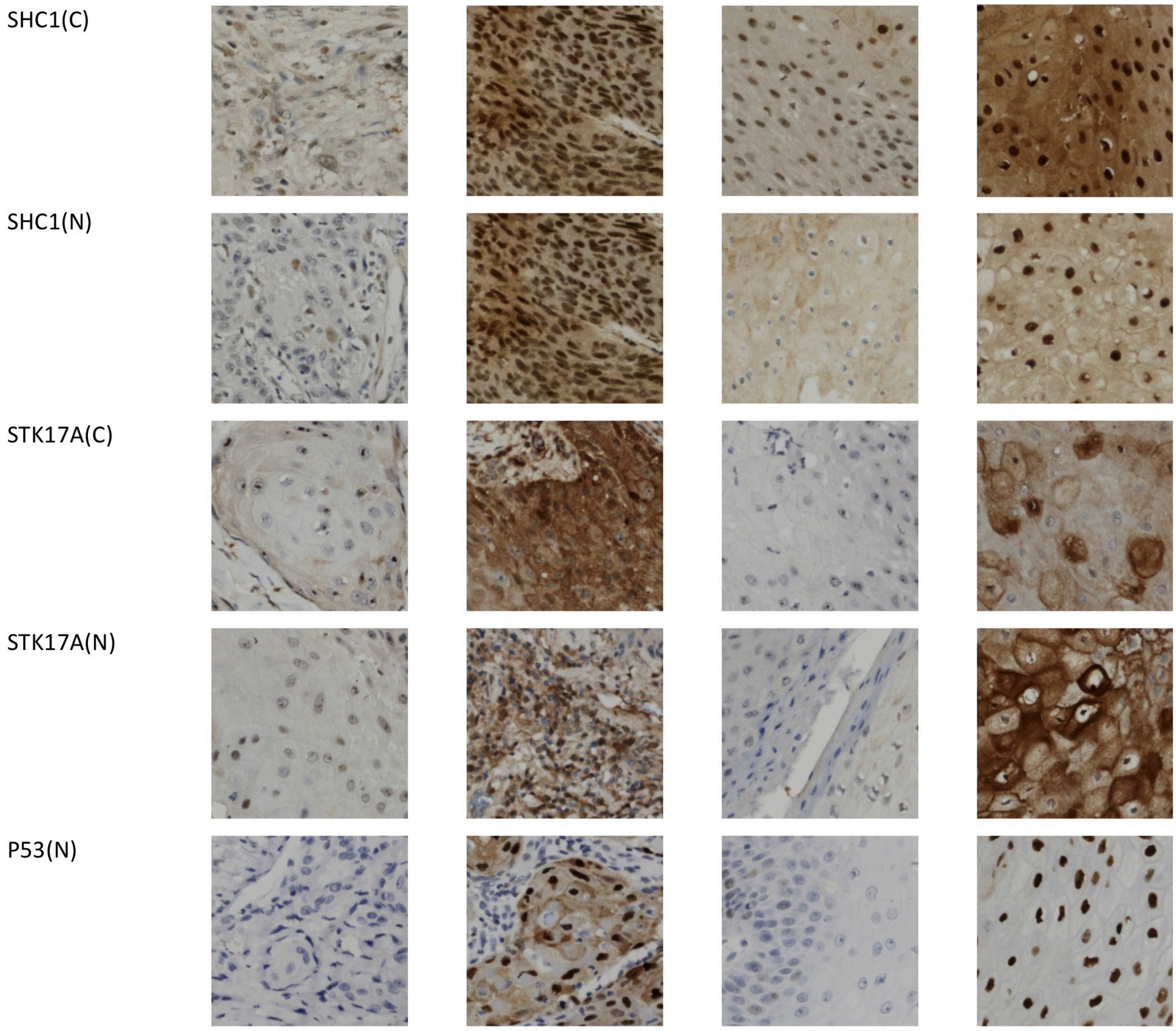
SHC1(C)
SHC1(N)
STK17A(C)
STK17A(N)
P53(N)

Additional information:

RB1(N)	
(A) Score 0 (Negative) 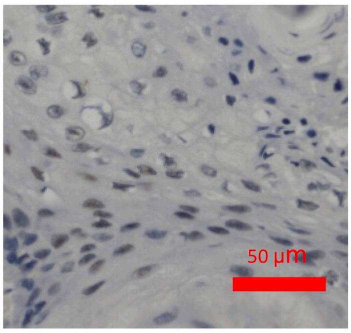	(B) Score 1+ 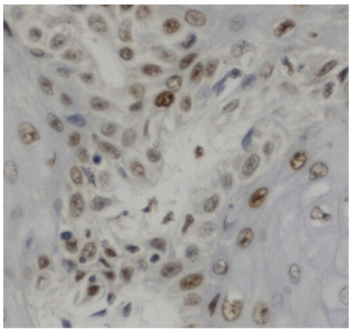
(C) Score 2+ 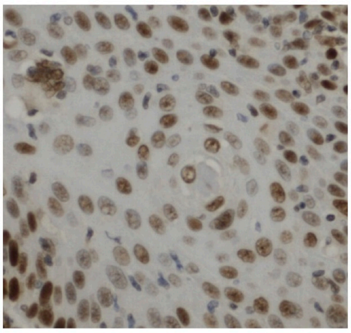	(D) Score 3+ 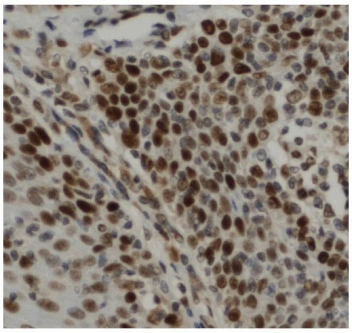

Note:

IHC staining was classified into four categories. (A) score 0 (negative) indicates very faint or absent staining, (B) score 1+ indicates weak staining, (C) score 2+ indicates moderate staining, and (C) score 3+ indicates strong staining based on staining intensity.
