# Supplementary material for: Immunohistochemical Expression of Five Protein Combinations Revealed as Prognostic Markers in Asian Oral Cancer
Source: Front Genet. 2021 Apr 15;12:643461. doi: 10.3389/fgene.2021.643461 (PMC8083901; doi:10.3389/fgene.2021.643461)
Supplement: Supplementary file 2 [file Table_1.pdf]

Table S1. The antibodies and retrieval buffers for each protein.

Protein Name	Clonality	Source	Catalogue number	Dilution	Retrieval buffer
BRCA1	Mouse monoclonal	Zeta Corporation	Z2237	1:100	Tris-EDTA buffer
CDH3	Rabbit polyclonal	Abgent	AP1499B	1:50	Tris-EDTA buffer
CDK6	Rabbit monoclonal	Abcam Ltd	ab124821	1:100	Tris-EDTA buffer
CSNK1E	Rabbit polyclonal	Abgent	AP7403a	1:50	Tris-EDTA buffer
EGFR	Rabbit monoclonal	Zeta Corporation	Z2037	1:50	Tris-EDTA buffer
FEN1	Rabbit polyclonal	Abcam Ltd	ab70815	1:1000	Tris-EDTA buffer
FLNA	Rabbit polyclonal	Abgent	AP7770a	1:50	Tris-EDTA buffer
KRAS	Rabbit polyclonal	Abcam Ltd	ab216890	1:200	Citrate buffer
MET	Rabbit polyclonal	Abgent	AP3167a	1:50	Citrate buffer
MSH2	Mouse monoclonal	Zeta Corporation	Z2129	1:100	Tris-EDTA buffer
P16	Mouse monoclonal	BD biosciences	550834	1:100	Tris-EDTA buffer
PARP1	Rabbit monoclonal	Abcam Ltd	Ab191217	1:500	Tris-EDTA buffer
PIM1	Rabbit polyclonal	Abgent	AP7932d	1:50	Tris-EDTA buffer
PLK1	Rabbit polyclonal	Abgent	AP7937a	1:100	Citrate buffer
POLB	Rabbit polyclonal	Abgent	AP50642	1:100	Tris-EDTA buffer
RAD54B	Rabbit polyclonal	Genetex	GTX103291	1:500	Tris-EDTA buffer
RB1	Mouse monoclonal	Leica Biosystems	NCL-L-RB-358	1:50	Tris-EDTA buffer
SGK2	Rabbit polyclonal	Abgent	AP7947b	1:100	Citrate buffer
SHC1	Rabbit polyclonal	Abgent	AP50024	1:100	Citrate buffer
STK17A	Rabbit polyclonal	Abcam Ltd	ab97530	1:100	Citrate buffer
TP53	Mouse monoclonal	Leica Biosystems	NCL-L-p53-DO7	1:200	Citrate buffer
