# Supplementary material for: Immunohistochemical Expression of Five Protein Combinations Revealed as Prognostic Markers in Asian Oral Cancer
Source: Front Genet. 2021 Apr 15;12:643461. doi: 10.3389/fgene.2021.643461 (PMC8083901; doi:10.3389/fgene.2021.643461)
Supplement: Supplementary file 3 [file Table_2.docx]

**Table S2. The scoring criteria of previous studies and references.**

| **Genes** | **Functions** | **Expression** | **References** |
| --- | --- | --- | --- |
| BRCA1 | - Tumor suppressor genes - DNA damage repair | Under-expression | ^5^ |
| CDH3(Placental-Cadherin) | - Cell-cell adhesion | Over- expression | ^15^ |
| CDK6 | - Regulation of cell cycle and differentiation | Over- expression | ^11^ |
| CSNK1E (Casein kinase 1 epsilon) | - DNA damage repair - Regulation of cell cycle and differentiation - Cell cytokinesis, differentiation, and apoptosis regulators | Under- expression | ^6^ |
| EGFR | - Cell proliferation, migration, metastasis, apoptosis, and angiogenesis | Over- expression | ^17^ |
| FEN1(DNA ﬂap endonuclease 1) | - DNA damage repair, including BER, HR and NER | Over- expression | ^7^ |
| FLNA | - Cell-cell adhesion | Under- expression | ^16^ |
| KRAS | - Oncogene - Signal transduction - Regulation of cell cycle and differentiation | Over- expression | ^1,3^ |
| MET | - Cell proliferation, migration, survival, angiogenesis, and invasion - Hepatocyte growth factor signaling pathway | Under- expression | ^18^ |
| MSH2 | - DNA damage repair | Under-expression | ^8^ |
| PARP1 | - DNA damage repair | Over-expression | ^21^ |
| P16 | - Regulation of cell cycle and differentiation | Over-expression | ^12^ |
| PIM1(*p*rovirus *i*ntegration site for *M*oloney murine leukemia virus *1*) | - Regulation of cell cycle and differentiation - Transcriptional regulators and signal transduction - Cell apoptosis regulators | Over-expression | ^13^ |
| PLK1 | - Regulation of cell cycle and differentiation | Over-expression | ^14^ |
| POLB (DNA polymerase beta) | - DNA damage repair | (N) Under- expression,  (C) Over-expression | ^5^, ^9^ |
| RAD54B | - DNA damage repair | Over-expression | ^10^ |
| RB1 | - Tumor suppressor genes - Regulation of cell cycle and differentiation | Over-expression | ^4^ |
| SGK2 | - Oncogenes - Modulation of β-catenin/c-Myc expression - Cell proliferation | Over-expression | ^20^ |
| SHC1(p66Shc) | - Signal transduction to EGFR pathways | Under- expression | ^5^ |
| STK17A | - Cell apoptosis regulators - Regulation of p53 | Over- expression | ^19^ |
| TP53 | - Tumor suppressor genes - DNA repair - Regulation of cell cycle and differentiation | Over- expression | ^2^ |

References:

1 Bruckman, K. C., Schönleben, F., Qiu, W., Woo, V. L. & Su, G. H. Mutational analyses of the BRAF, KRAS, and PIK3CA genes in oral squamous cell carcinoma. *Oral Surgery, Oral Medicine, Oral Pathology, Oral Radiology, and Endodontology* **110**, 632-637 (2010).

2 Lindemann, A., Takahashi, H., Patel, A., Osman, A. A. & Myers, J. N. Targeting the DNA Damage Response in OSCC with TP 53 Mutations. *Journal of dental research* **97**, 635-644 (2018).

3 Oikonomou, E., Koustas, E., Goulielmaki, M. & Pintzas, A. BRAF vs RAS oncogenes: are mutations of the same pathway equal? Differential signalling and therapeutic implications. *Oncotarget* **5**, 11752 (2014).

4 Thomas, S., Balan, A. & Balaram, P. The expression of retinoblastoma tumor suppressor protein in oral cancers and precancers: A clinicopathological study. *Dental research journal* **12**, 307 (2015).

5 Thul, P. J., & Cecilia L. The human protein atlas: A spatial map of the human proteome. *Protein Science*. **27**: 233-244 (2018).

6 Lin, S.H. *et al.* Casein kinase 1 epsilon expression predicts poorer prognosis in low T-stage oral cancer patients. *International journal of molecular sciences* **15**, 2876-2891 (2014).

7 Satapathy, S. R., Siddharth, S., Das, D., Nayak, A. & Kundu, C. N. Enhancement of cytotoxicity and inhibition of angiogenesis in oral cancer stem cells by a hybrid nanoparticle of bioactive quinacrine and silver: implication of base excision repair cascade. *Molecular pharmaceutics* **12**, 4011-4025 (2015).

8 Pereira, C. S. *et al.* Low expression of MSH2 DNA repair protein is associated with poor prognosis in head and neck squamous cell carcinoma. *Journal of Applied Oral Science* **21**, 416-421 (2013).

9 Maitra, A. *et al.* Mutational landscape of gingivo-buccal oral squamous cell carcinoma reveals new recurrently-mutated genes and molecular subgroups. *Nature communications* **4**, 2873 (2013).

10 Wright, W. D. & Heyer, W.-D. Rad54 functions as a heteroduplex DNA pump modulated by its DNA substrates and Rad51 during D loop formation. *Molecular cell* **53**, 420-432 (2014).

11 Andisheh-Tadbir, A., Ashraf, M. J. & Jeiroodi, N. Expression of CDK6 in oral squamous cell carcinomas. *Asian Pacific journal of cancer prevention: APJCP* **19**, 1013 (2018).

12 Sritippho, T., Chotjumlong, P. & Iamaroon, A. Roles of human papillomaviruses and p16 in oral cancer. *Asian Pac J Cancer Prev* **16**, 6193-6200 (2015).

13 Chen, J. & Tang, G. PIM-1 kinase: a potential biomarker of triple-negative breast cancer. *OncoTargets and therapy* **12**, 6267 (2019).

14 Liu, Z., Sun, Q. & Wang, X. PLK1, a potential target for cancer therapy. *Translational oncology* **10**, 22-32 (2017).

15 Milicic, A. *et al.* Ectopic expression of P-cadherin correlates with promoter hypomethylation early in colorectal carcinogenesis and enhanced intestinal crypt fission in vivo. *Cancer research* **68**, 7760-7768 (2008).

16 Zhang, Y. *et al.* FLNa negatively regulated proliferation and metastasis in lung adenocarcinoma A549 cells via suppression of EGFR. *Acta biochimica et biophysica Sinica* **50**, 164-170 (2018).

17 Kimura, I. *et al.* Loss of epidermal growth factor receptor expression in oral squamous cell carcinoma is associated with invasiveness and

18 Sun, Z. *et al.* Role of c-Met in the progression of human oral squamous cell carcinoma and its potential as a therapeutic target. *Oncology reports* **39**, 209-216 (2018).

19 Park, Y. *et al.* Cytoplasmic DRAK1 overexpressed in head and neck cancers inhibits TGF-β1 tumor suppressor activity by binding to Smad3 to interrupt its complex formation with Smad4. *Oncogene* **34**, 5037-5045 (2015).

20 Baldwin, A. *et al.* Kinase requirements in human cells: V. Synthetic lethal interactions between p53 and the protein kinases SGK2 and PAK3. *Proceedings of the National Academy of Sciences* **107**, 12463-12468 (2010).

21 Kossatz, S. *et al*. Detection and delineation of oral cancer with a PARP1 targeted optical imaging agent. *Scientific reports* **6**: 21371 (2016).
