# Supplementary material for: Immunohistochemical Expression of Five Protein Combinations Revealed as Prognostic Markers in Asian Oral Cancer
Source: Front Genet. 2021 Apr 15;12:643461. doi: 10.3389/fgene.2021.643461 (PMC8083901; doi:10.3389/fgene.2021.643461)
Supplement: Supplementary file 4 [file Table_3.docx]

**Table S3.** Testing association between habits and IHC markers.

| 1. **Fisher test** | | |
| --- | --- | --- |
| **Habit: alcohol, betal nut chewing and smoking** | **Subset** | ***p*-value** |
| RB1(N) | ↑/↓^a^ | 0.185 |
| (CSNK1E(C), SHC1(N)) | (↓, ↓)/otherwise | 0.732 |
| (CSNK1E(C), RB1(N)) | (↓, ↑)/otherwise | 0.816 |
| (CDH3(C), STK17A(N)) | (↑, ↑)/otherwise | 1.000 |
| (BRCA1(N), SHC1(N)) | (↓, ↓)/otherwise | 0.512 |
| (FLNA(C), KRAS(C)) | (↑, ↑)**^c^**/otherwise | 0.283 |
| [CSNK1E(C)-SHC1(N), FLNA(C)-KRAS(C)] | [(↓, ↓), (↑, ↑)**^c^**] /otherwise | 0.702 |
| [BRCA1(N)-SHC1(N), FLNA(C)-KRAS(C)] | [(↓, ↓), (↑, ↑)**^c^**]/otherwise | 0.731 |

| 1. **Fisher test** | | |
| --- | --- | --- |
| **Habit: alcohol use** | **Subset** | ***p*-value** |
| RB1(N) | ↑/↓^a^ | 0.490 |
| (CSNK1E(C), SHC1(N)) | (↓, ↓)/otherwise | 1.000 |
| (CSNK1E(C), RB1(N)) | (↓, ↑)/otherwise | 0.810 |
| (CDH3(C), STK17A(N)) | (↑, ↑)/otherwise | 0.651 |
| (BRCA1(N), SHC1(N)) | (↓, ↓)/otherwise | 0.743 |
| (FLNA(C), KRAS(C)) | (↑, ↑)**^c^***/otherwise | 0.355 |
| [CSNK1E(C)-SHC1(N), FLNA(C)-KRAS(C)] | [(↓, ↓), (↑, ↑)**^c^**] /otherwise | 1.000 |
| [BRCA1(N)-SHC1(N), FLNA(C)-KRAS(C)] | [(↓, ↓), (↑, ↑)**^c^**]/otherwise | 1.000 |
|  | | |

^a^The symbols “↑” and “↓” denote over- and under-expression of IHC, respectively of the corresponding protein.

**^*^**The symbol (↑, ↑)^c^ denotes the complementary set of (↑, ↑), namely (↓, ↑), (↑, ↓) and (↓, ↓), in which FLNA(C)-KRAS(C) is in the same direction (poor OS) as that of BRCA1(N)-SHC1(N).
