# Supplementary material for: Immunohistochemical Expression of Five Protein Combinations Revealed as Prognostic Markers in Asian Oral Cancer
Source: Front Genet. 2021 Apr 15;12:643461. doi: 10.3389/fgene.2021.643461 (PMC8083901; doi:10.3389/fgene.2021.643461)
Supplement: Supplementary file 5 [file Table_4.pdf]

Table S4a Multivariate Cox Regression

**Variable**	**Subset**	**Hazard ratio (95% CI)**	***p*-value**	$LR_{\chi 2}$
RB1(N)	↑/↓	1.68 (0.87 - 3.24)	0.122	18.3
Stage	III–IV/I–II	2.13 (0.86 - 5.27)	0.104	
Lymph node metastasis	yes/no	1.86 (0.78 - 4.47)	0.163	
(CSNK1E(C), SHC1(N))	(↓, ↓)/otherwise	8.89 (3.22 - 24.55)	0.000	25.6
Stage	III–IV/I–II	2.28 (0.91 - 5.76)	0.080	
Lymph node metastasis	yes/no	1.96 (0.8 - 4.85)	0.143	
(CSNK1E(C), RB1(N))	(↓, ↑)/otherwise	2.13 (1.01 - 4.53)	0.048	18.6
Stage	III–IV/I–II	2.01 (0.81 - 4.99)	0.133	
Lymph node metastasis	yes/no	1.90 (0.79 - 4.54)	0.151	
(BRCA1(N), SHC1(N))	(↓, ↓)/otherwise	2.7 (1.04 - 7.01)	0.042	18.3
Stage	III–IV/I–II	2.45 (0.98 - 6.15)	0.056	
Lymph node metastasis	yes/no	1.63 (0.67 - 3.98)	0.286	

Table S4b Multivariate Cox Regression on combinations of two significant markers

**Variable**	**Hazard ratio (95% CI)**	***p*-value**	$LR_{\chi 2}$
CSNK1E(C)-SHC1(N) (↓, ↓) and FLNA(C)-KRAS(C) (↑, ↑)[c*]	12.76 (3.95 - 41.21)	0.000	23.12
Stage	1.62 (0.59 - 4.44)	0.348	
Lymph node metastasis	2.64 (0.96 - 7.28)	0.060	
BRCA1(N)-SHC1(N) (↓, ↓) and FLNA(C)-KRAS(C)(↑, ↑)[c]	2.91 (1.1 - 7.68)	0.031	16.08
Stage	1.99 (0.75 - 5.32)	0.169	
Lymph node metastasis	1.81 (0.7 - 4.69)	0.223	

[*]The symbol (↑, ↑)[c] denotes the complementary set of (↑, ↑), namely (↓, ↑), (↑, ↓) and (↓, ↓), in which FLNA(C)-KRAS(C) is in the same direction (poor OS) as that of BRCA1(N)-SHC1(N).
